# Transient T cell depletion causes regression of melanoma metastases

**DOI:** 10.1186/1479-5876-6-12

**Published:** 2008-03-11

**Authors:** Mary Ann Rasku, Amy L Clem, Sucheta Telang, Beverly Taft, Kelly Gettings, Hana Gragg, Daniel Cramer, Sheron C Lear, Kelly M McMasters, Donald M Miller, Jason Chesney

**Affiliations:** 1 Molecular Targets Program, James Graham Brown Cancer Center, University of Louisville School of Medicine, Louisville, KY, USA; 2 Department of Pathology, University of Louisville School of Medicine, Louisville, KY, USA; 3 Department of Surgery, University of Louisville School of Medicine, Louisville, KY, USA

## Abstract

**Background:**

Cognate immunity against neoplastic cells depends on a balance between effector T cells and regulatory T (Treg) cells. Treg cells prevent immune attack against normal and neoplastic cells by directly suppressing the activation of effector CD4^+ ^and CD8^+ ^T cells. We postulated that a recombinant interleukin 2/diphtheria toxin conjugate (DAB/IL2; Denileukin Diftitox; Ontak) may serve as a useful strategy to deplete Treg cells and break tolerance against neoplastic tumors in humans.

**Methods:**

We administered DAB/IL2 (12 μg/kg; four daily doses; 21 day cycles) to 16 patients with metastatic melanoma and measured the effects on the peripheral blood concentration of several T cell subsets and on tumor burden.

**Results:**

We found that DAB/IL2 caused a transient depletion of Treg cells as well as total CD4^+ ^and CD8^+ ^T cells (< 21 days). T cell repopulation coincided with the *de novo *appearance of melanoma antigen-specific CD8^+ ^T cells in several patients as determined by flow cytometry using tetrameric MART-1, tyrosinase and gp100 peptide/MHC conjugates. Sixteen patients received at least one cycle of DAB/IL2 and five of these patients experienced regressions of melanoma metastases as measured by CT and/or PET imaging. One patient experienced a near complete response with the regression of several hepatic and pulmonary metastases coupled to the *de novo *appearance of MART-1-specific CD8^+ ^T cells. A single metastatic tumor remained in this patient and, after surgical resection, immunohistochemical analysis revealed MART1^+ ^melanoma cells surrounded by CD8^+ ^T cells.

**Conclusion:**

Taken together, these data indicate that transient depletion of T cells in cancer patients may disrupt the homeostatic control of cognate immunity and allow for the expansion of effector T cells with specificity against neoplastic cells. Several T cell depleting agents are clinically available and this study provides strong rationale for an examination of their efficacy in cancer patients.

**Trial registration:**

NCT00299689 (ClinicalTrials.gov Identifier).

## Background

Of the common adult-onset cancers, melanoma is widely held to be the most amenable to immunotherapeutic intervention. This perception is based on the following: (i) several melanoma-specific antigens have been identified; (ii) melanoma antigen-specific CD4^+ ^and CD8^+ ^T lymphocytes are increased in melanoma patients and have anti-tumor activity; (iii) immune-enhancing agents can cure mice of established melanomas; and (iv) spontaneous melanoma regressions in humans with the simultaneous onset of vitiligo have been reported [1,2]. Additionally, the immune-enhancing agent, interferon-α, increases survival in melanoma patients with intermediate or high risk of recurrence, and high-dose interleukin 2 (IL-2), a potent stimulator of T cell proliferation, causes durable remissions in a small subset of patients with metastatic melanoma [3-5].

Cognate immune reactions against tumor cells depend on a balance between activated tumor antigen specific CD4^+ ^and CD8^+ ^T cells and suppressive regulatory T cells [6]. CD4^+^CD25^HI ^regulatory T (Treg) cells directly suppress the activation of anti-tumor effector T cells in a contact-dependent manner [7,8]. Depletion of these immunosuppressive Treg cells using anti-CD25 monoclonal antibodies enables a CD8^+ ^and CD4^+ ^T cell dependent immune rejection against the progression of melanoma in mice [7,9,10]. This anti-tumor activity is not exclusive to melanoma since it has been observed against several transplantable mouse tumor cell lines in multiple disparate mouse strains [11-13]. Furthermore, down-regulation of Treg cell function can be accomplished with certain stimulatory antibodies (*e.g*. anti-GITR [DTA-1]) and this treatment confers concomitant tumor immunity against remote tumors even in poorly immunogenic mouse melanomas [10]. Treg cells thus may function to tolerize or deactivate cognate effector CD4^+ ^and CD8^+ ^T cells to neoplastic cells that express tumorigenic antigens. Accordingly, the selective depletion of these cells may have clinical utility for the induction of anti-tumor immunity.

A multitude of successful treatment strategies in tumor-bearing mice have failed when examined in human clinical trials, and correlative data that provide additional rationale for clinical trials in humans is essential. Viguier *et al*. reported the results of an immunohistochemical examination for the presence of CD4^+^CD25^HI ^Treg cells in lymph nodes with and without metastasic melanoma seeds extracted from twelve patients with metastatic melanoma (Stage IIIb/IIIc) [14]. They observed that the frequency of Treg cells in melanoma-positive lymph nodes was 11.06% whereas the frequency was only 6.2% in melanoma-free lymph nodes [14]. The over-representation of Treg cells in metastatic lymph nodes suggests that these cells may be recruited and/or expanded at the tumor site. Treg cells isolated from the metastatic lymph nodes were found to inhibit CD4^+ ^and CD8^+ ^T cell proliferation *in vitro *via a contact-dependent mechanism and thus appear to function similarly to the well characterized mouse Treg cells. Taken together, these data provide correlative evidence that CD25^+ ^Treg cells may be functioning to suppress anti-tumor immunity in melanoma patients.

Denileukin diftitox (DAB/IL2; Ontak) is a recombinant DNA-derived cytotoxic protein composed of diphtheria toxin fragments A and B and the full-length IL-2 molecule. DAB/IL2 binds to CD25 (the α chain of the IL-2 receptor) and, following internalization, inhibits protein synthesis, causing cell death within hours [15]. DAB/IL2 is FDA-approved for the treatment of patients with persistent or recurrent cutaneous T cell lymphoma (CTCL) whose malignant cells express CD25. CTCL malignant proliferation is driven by interaction with cutaneous dendritic cells (termed Langerhans cells), and this epidermal association of CTCL cells with Langerhans cells is a hallmark of the malignancy [16]. In recent studies, purified human CTCL cells were incubated with dendritic cells and found to adopt a Treg phenotype, including: (i) expression of the negative T cell regulator, cytotoxic T lymphocyte antigen-4 (CTLA-4); (ii) expression of the Treg specific transcription factor, Foxp3; and (iii) the ability to suppress CD4^+ ^T cell activation [16]. Based on these findings, CTCL has been postulated to be a malignant proliferation of Treg cells and, coupled to the observation that Treg cells express high surface CD25, these data provide rationale for the testing of DAB/IL2 as a selective Treg-depleting agent.

In the current study, we have found that DAB/IL2 administration to stage IV melanoma patients depletes peripheral blood Treg cells and causes the regression of metastatic tumors in a subset of patients. We also observed a significant depletion of the total T cell population including CD4^+^CD25^- ^T cells that was reversed within 21 days of DAB/IL2 administration. We speculate that transient depletion of T cells in cancer patients may disrupt the homeostatic control of cognate immunity and allow an expansion of CD8^+ ^T cells with increased specificity for the peptide/MHC complexes expressed by neoplastic cells.

## Methods

### Patient enrollment

This clinical trial was approved by the University of Louisville Human Studies Committee. Only patients with distant metastases from cutaneous or mucosal melanoma or melanoma of unknown primary were eligible for inclusion. All patients fulfilled the following criteria: (i) primary tumor must have been documented by histopathologic analysis; (ii) metastatic disease must have been documented by radiologic examinations (CT scan or PET scan) with bidimensional measurements; and (iii) disease recurrences occurring greater than five years after the original diagnosis must have been biopsy proven.

### DAB/IL2 administration

All patients were subjected to fusion PET/CT or CT imaging within one month prior to receiving the first dose of DAB/IL2 and within one month after receiving the last dose of DAB/IL2. DAB/IL2 was administered as follows: 12 μg/kg, IV over 30 minutes every 24 hours for 4 doses (cycles repeated every 21 days). All patients had renal function tests, blood counts, and a thorough physical examination, including neurological examination, prior to each cycle of DAB/IL2. The endpoint definitions were as follows:

#### Clinical complete response (CR)

Disappearance of all evidence of tumor. The patient must be free of all symptoms of cancer.

#### Partial response (PR)

30% or greater decrease in the sum of the longest diameter of target lesions, taking as reference the baseline sum longest diameter.

#### Progressive disease (PD)

At least 20% increase in the sum of the longest diameter of target lesions, taking as reference the baseline sum longest diameter, or the appearance of new lesions and/or unequivocal progression of existing non-target lesion.

#### Stable disease (SD)

Neither sufficient shrinkage to qualify for partial response nor sufficient increase to qualify for progressive disease, taking as reference the smallest sum longest diameter since the treatment started.

### Monocyte, granulocyte, lymphocyte and T cell subset quantification

Whole blood (50 ml) was collected in heparinized tubes and the absolute lymphocyte, granulocyte and monocyte peripheral blood concentrations were determined with a Sysmex XE-2100 Automated Hematology Analyzer. PBMCs were then isolated by centrifugation through Accuspin System Histopaque 1077 and washed twice with PBS.

In order to determine the percentage of CD4^+^, CD4^+^/CD25^-^, CD4^+^/CD25^+^, CD4^+^/CD25^HI^, CD4^+^/CD25^HI^/Foxp3^-^, and CD4^+^/CD25^HI^/Foxp3^+ ^T cells within the lymphocyte gate (based on forward/side scatter profile), we incubated the total PBMCs with PE-anti-Foxp3, FITC-anti-CD4, and APC-anti-CD25 (eBioscience). 100 μl of PBMCs (1 × 10^6^) were added to 20 μl of an anti-CD4/and-CD25 cocktail (1 μg anti-CD4 and 0.125 μg anti-CD25; eBioscience) and incubated for 30 minutes in the dark at 4°C and then washed in cold PBS. After decanting, the cell pellet was resuspended in residual buffer and 1 ml of freshly prepared eBioscience Fixation/Permeabilization Buffer was added to each sample and incubated at 4°C for 60 minutes in the dark. 2 ml of Permeabilization Buffer was used for washing followed by centrifugation and decanting of supernatant. 20 μl anti-human Foxp3 (PCH101) antibody or 20 μl rat IgG2b isotype control was added to resuspended cells and incubated at 4°C for 30 minutes in the dark. Cells were washed twice in 2 ml Permeabilization Buffer. Small lymphocytes were gated according to forward/side-scatter profiles and data was collected on a FACSCalibur flow cytometer within 1 hour after staining, and then analyzed with Cell Quest software (Becton Dickinson).

In order to detect the percentage of total CD8^+ ^cells, and MART1-, gp100- and tyrosinase-specific CD8+ T cells within the lymphocyte gate (based on forward/side scatter profile), 10^6 ^PBMCs in 200 μl of flow cytometry staining buffer were incubated at 25°C for 30 minutes in the dark with 1.0 μg of APC-labeled tetramer (MART-1, gp100 or tyrosinase; Immunomics, Beckman Coulter) and 0.25 μg CD8-PE monoclonal antibody (R&D Systems). Small lymphocytes were gated according to forward/side-scatter profiles and then the percentage of tetramer^+^CD8^+ ^cells was determined. Data was collected on a FACSCalibur flow cytometer within 1 hour after staining, and analyzed with Cell Quest software (Becton Dickinson).

The absolute concentrations of CD4^+^, CD4^+^/CD25^-^, CD4^+^/CD25^+^, CD4^+^/CD25^HI^, CD4^+^/CD25^HI^/Foxp3^-^, CD4^+^/CD25^HI^/Foxp3^+^, CD8^+^, CD8^+^/HLA-A2*0201-MART1-binding, CD8^+^/HLA-A2*0201-gp100-binding and CD8^+^/HLA-A2*0201-tyrosinase-binding cells were quantified by determining the percentage of fluorescence-positive cells within the forward/side scatter lymphocyte gate (as detailed above), and then multiplying this percentage by the absolute lymphocyte concentration determined using the Sysmex XE-2100 Automated Hematology Analyzer. The percent control of each sample was calculated by dividing the T cell subset absolute cell concentration on the indicated day of treatment with the cell concentration on day 0 prior to DAB/IL2 administration (× 100).

### DAB/IL2 enzyme linked immunosorbent assay

Human plasma samples were tested for the presence of IgG specific for DAB/IL2 by enzyme linked immunosorbent assay (ELISA). The assay was carried out as follows: 96-well microtest polystyrene assay plates (BD) were coated (100 μL/well) with either Tris-NaCl pH 8.5 solution (30 mL 5 M NaCl + 50 mL 1 M Tris + 920 mL water) or DAB/IL2 (Ligand) diluted to 2 μg/mL in Tris-NaCl solution. After incubating overnight at 37°C, the plates were washed two times with Tris-NaCl solution. 300 μl PBS/BSA (30 mL PBS + 300 mg BSA; Sigma) was then added to each well and the plates were incubated for one hour at 37°C, followed by three washes with Tris-NaCl solution. 100 μl of test sera, diluted 1:500 in PBS/BSA solution, was then added to each well. After incubating at 37°C for two hours, the plates were washed three times with Tris-NaCl + 0.05% Tween (300 mL Tris-NaCl + 150 μl Tween). 100 μl of rabbit anti-human IgG HRP-conjugated antibody (Pierce), diluted 1:50,000 in PBS/BSA, was then added to each well and the plates incubated at 37°C for one hour, followed by three washes with Tris-NaCl + 0.05% Tween and two washes with DH_2_O. 100 μl of TMB substrate (Pierce) was added to each well. After five minutes, the reaction was stopped with 1N HCL (100 μl/well) and the plates were read at 450 nm.

### Immunohistochemistry

Five μm sections of formalin-fixed and paraffin-embedded tumor tissue were mounted on charged glass slides and dried at 58°C for 60 minutes. Slides were first deparaffinized with xylene then incubated with a high temperature epitope retrieval solution (20 min) and hydrogen peroxide (H_2_O_2_) (for 10 min) to block endogenous peroxidases. The sections were incubated with primary antibody (anti-CD8, 1:50, Dako; anti-CD4, 1:50, Novocastra; anti-HLA Class I [HLA-A, B, C], 1:500, clone EMR8-5, MBL International) for 15 min, followed by a post-primary antibody and a polymer horse-radish-peroxidase linked detection system (each for 8 min, Define, Leica Microsystems). The sections were developed with 3,3'-diaminobenzidine tetrahydrochloride (DAB) solution (Invitrogen) for 10 min and nuclei counterstained with hematoxylin (Dako) for 7 min. PBS washes were performed between all steps. The slides were neutralized in ammonia water, dehydrated in graded alcohols (100%, 95%, and 80% ethanol [vol/vol] in H_2_O), cleared in xylene and coverslips attached with Permount (Fisher Scientific).

For MART-1 staining, slides were deparaffinized (with xylene), hydrated with distilled water and then placed in citrate buffer (Dako) in a 72°C oven overnight for antigen retrieval. Following treatment with H_2_O_2_, slides were incubated in MART-1 primary antibody (1:40, Signet) for 25 min then in LSAB2 biotinylated link antibody (Dako) for 20 min followed by a streptavidin-peroxidase reaction using DAB as a chromogen. Slides were finally counterstained in hematoxylin and then neutralized, dehydrated and coverslips attached as above. Double staining was accomplished by first staining for CD8 (as above) using DAB as the chromogen followed by washing and staining for MART-1 using the alkaline phosphatase system (Leica) omitting the deparaffinization and retrieval steps. Brown staining from the DAB indicated CD8^+ ^T cells and red staining from the alkaline phosphatase indicated MART1^+ ^cells. Both positive and negative controls were stained with the specimens.

### Cytotoxicity assay

CRL-11174 human melanoma cells (ATCC) were cultured in 1 ml of Dulbecco's Modified Eagle Medium (DMEM) (Hyclone, Logan, UT) supplemented with 10% fetal bovine serum (FBS) (Hyclone, Logan, UT) and 50 μg/mL gentamicin sulfate (Invitrogen, Carlsbad, CA) (2.5 × 10^5 ^cells/well, 6-well plate). DAB/IL2 (Ligand Pharmaceuticals) or PBS was added to the culture (0.05–5 μg/ml) and, after 48 hours, live and dead cells were enumerated by the addition of trypan blue and direct visualization using light microscopy.

## Results

### DAB/IL2 transiently depletes CD4^+^, CD8^+ ^and CD4^+^/CD25^+^/Foxp3^+ ^T cells

**Figure 1:**
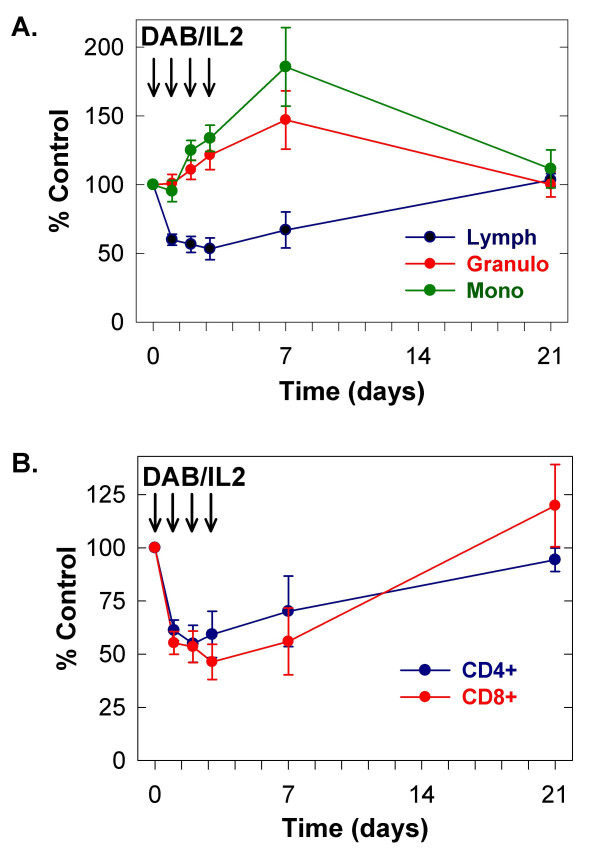
**DAB/IL2 transiently depletes CD4^+ ^and CD8^+ ^T cells in melanoma patients**. 10 patients with stage IV metastatic melanoma were administered DAB/IL2 (intravenous; 12 μg/kg) daily × 4 days (arrows indicate each administration). Whole blood was collected on the indicated days and analyzed for absolute lymphocyte (black), granulocyte (red) and monocyte (green) concentration with an automated hematology analyzer (**A**) and absolute CD4^+ ^and CD8^+ ^T cell concentration by flow cytometry (**B**). The peripheral blood concentrations of CD4^+ ^and CD8^+ ^T cells were quantified by multiplying the percentage of anti-CD4 or anti-CD8 fluorescence-positive cells within the lymphocyte forward/side scatter gate by the absolute lymphocyte concentration determined using an automated hematology analyzer. Percent control of each sample was calculated by dividing the absolute cell concentration on the indicated day of treatment with the absolute cell concentration on day 0 prior to DAB/IL2 administration (× 100). Data are represented as averages ± standard error of the mean (n = 10 patients).

**Figure 2:**
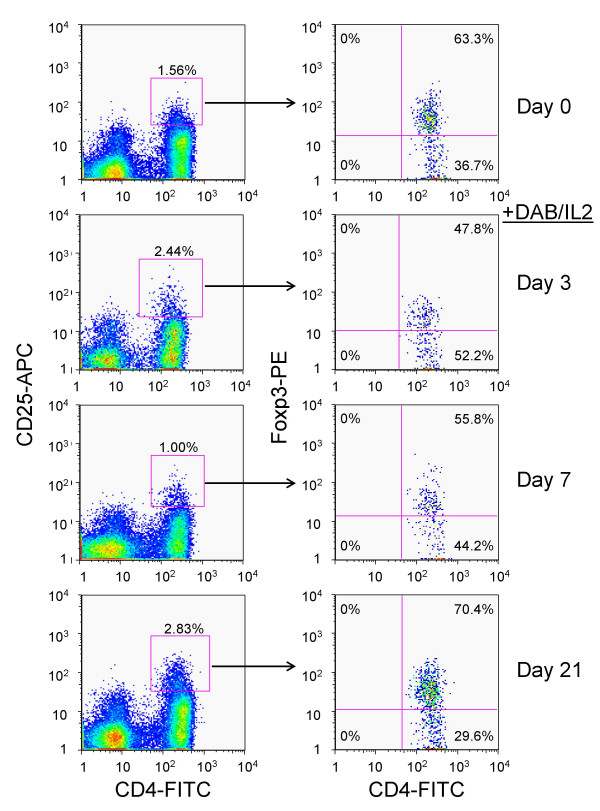
**DAB/IL2 transiently depletes CD4^+^/CD25^HI^/Foxp3^+ ^T cells**. Whole blood was collected from patient P9 during cycle one of DAB/IL2 administration just prior to the first (day 0) and last dose (day 3) and then 7 and 21 days after initiation of DAB/IL2 therapy. The peripheral blood mononuclear cells were isolated from the whole blood by Ficoll gradient centrifugation and stained with fluorescent conjugates of monoclonal antibodies specific for CD4, CD25 and Foxp3. In order to quantify the percentage of CD4^+^/CD25^HI^/Foxp3^+ ^T cells within the total lymphocyte forward/side scatter gate, the CD4^+^/CD25^HI ^cells (right panels) were gated and analyzed for Foxp3 expression (left panels).

**Figure 3:**
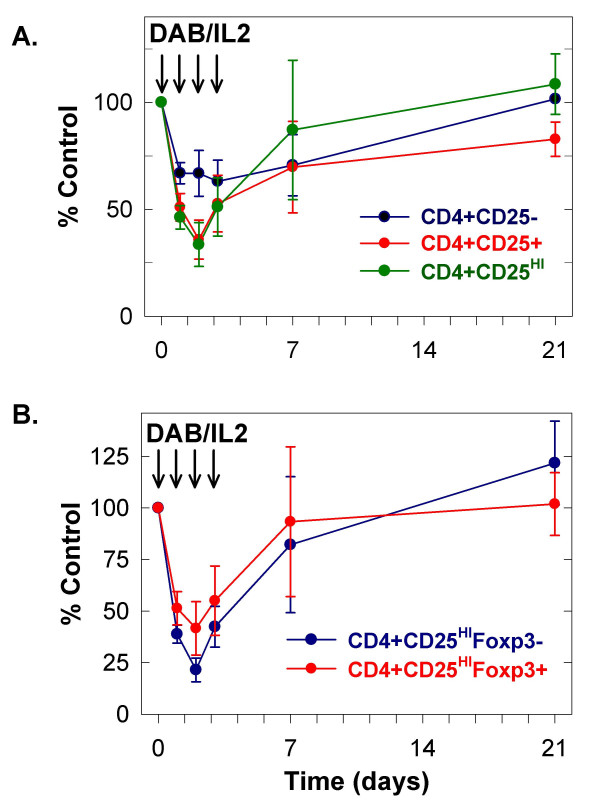
**DAB/IL2 transiently depletes all analyzed CD4^+ ^T cell subsets**. Whole blood was collected from 10 patients throughout the first cycle of DAB/IL2 and analyzed for CD4^+^/CD25^HI^/Foxp3^+ ^co-expression by flow cytometry as described in the Figure 2 legend and Methods section. The absolute concentration of CD4^+^/CD25^- ^(black), CD4^+^/CD25^+ ^(red) and CD4^+^/CD25^HI ^(green) T cells (**A**) and of CD4^+^/CD25^HI^/Foxp3^- ^(black) and CD4^+^/CD25^HI^/Foxp3^+ ^(red) T cells (**B**) were quantified by multiplying the percentage of anti-CD4, anti-CD25 and/or anti-Foxp3 fluorescence-positive cells within the lymphocyte forward/side scatter gate by the absolute lymphocyte concentration determined using an automated hematology analyzer. The percent control of each sample was calculated by dividing the absolute cell concentration on the indicated day of treatment with the cell concentration on day 0 prior to DAB/IL2 administration (× 100). Data are represented as averages ± standard error of the mean (n = 10 patients).

We administered DAB/IL2 (12 μg/kg daily × four days) to stage IV melanoma patients and measured the peripheral blood concentration of lymphocytes, granulocytes and monocytes on days 0, 1, 2, 3, 4, 7 and 21 using an automated hematology analyzer (Figure 1A; n = 10). We observed a reduction in the absolute lymphocyte concentration and an increase in granulocytes and monocytes within 48 hours of DAB/IL2 administration. Next, the absolute concentration of several T cell subsets was quantified by multiplying the percentage of fluorescence-positive cells within the lymphocyte forward/side scatter gate determined using flow cytometric analyses by the absolute lymphocyte concentration determined using an automated hematology analyzer. We quantified the absolute CD4^+ ^and CD8^+ ^T cell concentration in the peripheral blood and found that both T cell subsets were reduced to ~ 50% of control within 24–48 hours of DAB/IL2 administration (Figure 1B; n = 10). We co-stained for CD4, CD25 and Foxp3 expression and found that DAB/IL2 depleted the CD4^+^CD25^HI^Foxp3^+ ^cell percentage in the lymphocyte gate within 72 hours but that these CD4^+^CD25^HI^Foxp3^+ ^cells repopulated the peripheral blood within 21 days (see Figure 2 for a representative example; CD4^+^CD25^HI ^cells [left panels] were gated and examined for Foxp3 expression [right panels]). Co-staining for CD4, CD25 and Foxp3 allowed us to gate on several distinct T cell populations and calculate peripheral blood concentrations (based on the absolute lymphocyte concentrations) in order to identify the T cell phenotypes that are most sensitive to DAB/IL2 administration. We were surprised that the peripheral blood CD4^+^CD25^- ^T cell concentration was depleted by DAB/IL2 albeit to a lesser extent than CD4^+^CD25^+ ^and CD4^+^CD25^HI ^T cells (Figure 3A; n = 10; *p *value < 0.05 for each comparison). Given that the targeting mechanism of DAB/IL2 is through CD25, we can only speculate that the depletion of CD4^+^CD25^- ^cells is due to unidentified indirect consequences of CD4^+^CD25^+ ^T cell depletion. Interestingly, Foxp3 expression did not significantly alter sensitivity to DAB/IL2 since the depletion of CD4^+^CD25^HI^Foxp3^+ ^and CD4^+^CD25^HI^Foxp3^- ^was not statistically different (Figure 3B; *p *value = 0.424).

**Figure 4:**
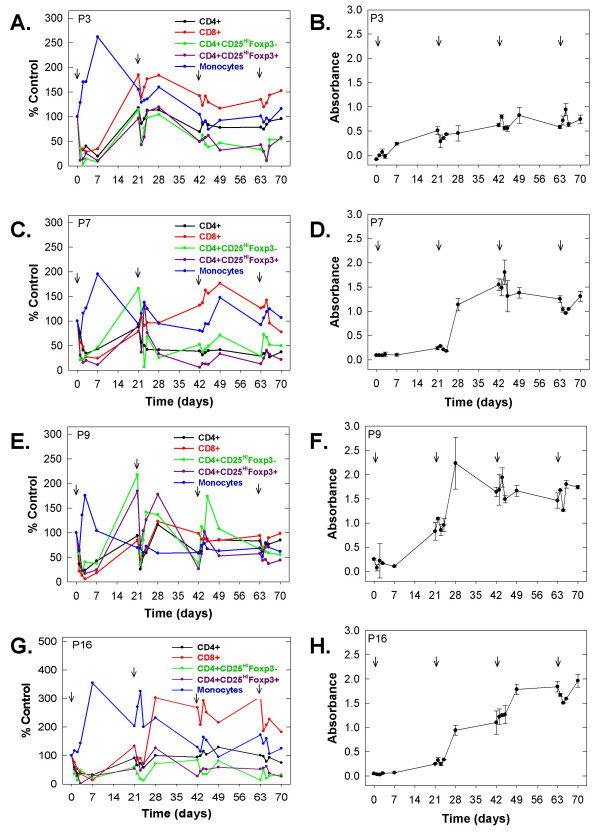
**Reduction in the T cell depleting activity of DAB/IL2 during cycles 2–4 is associated with the development of anti-DAB/IL2 IgG**. Whole blood was collected on the indicated days from patients P3 (**A, B**), P7 (**C, D**), P9 (**E, F**) and P16 (**G, H**) throughout four cycles of DAB/IL2 administration (each cycle indicated by an arrow). CD4^+ ^(black), CD8^+ ^(red), CD4^+^/CD25^HI^/Foxp3^- ^(green) and CD4^+^/CD25^HI^/Foxp3^+ ^(purple) T cells and monocytes (blue) were quantified as described in Figures 1 – 3 (**A**, **C**, **E**, **H**). Percent control of each sample was calculated by dividing the absolute cell concentration on the indicated day of treatment with the cell concentration on day 0 prior to DAB/IL2 administration (× 100). Plasma was isolated on the indicated days and analyzed for the presence of anti-DAB/IL2 IgG by ELISA. For the ELISA, data are presented as averages ± standard deviations (n = 5 per sample).

In four patients that received four 3-week cycles (P3, P7, P9 and P16), we found that the T cell depletion and inverse increase in peripheral blood monocytes was blunted in cycles 2–4 of DAB/IL2 (Figures 4A, 4C, 4E, and 4G; arrows indicate cycles) and that this effect was coincident with the appearance of anti-DAB/IL2-specific IgG as measured by ELISA (Figures 4B, 4D, 4F, and 4H). Additionally, we found that the CD4^+^CD25^HI^Foxp3^+ ^T cells were depleted to a greater extent relative to CD8^+ ^T cells after 4 cycles of DAB/IL2 administration (day 70; CD4^+^CD25^HI^Foxp3^+ ^T cells = 37 ± 16% of control; CD8+ T cells, 128 ± 48% of control; n = 4; *p *value = 0.031; Figures 4A, 4C, 4E, and 4G). Although the total CD4^+ ^T cell recovery after 4 cycles of DAB/IL2 appeared not to be as robust as that observed by the CD8^+ ^T cells, we observed no statistically significant difference in these T cell subsets (CD4^+ ^T cells, 73 ± 25% of control; CD8+ T cells, 128 ± 48% of control; n = 4; *p *value = 0.086).

### T cell repopulation after DAB/IL2 monotherapy is associated with the de novo appearance of melanoma antigen-specific CD8^+ ^T cells

**Figure 5:**
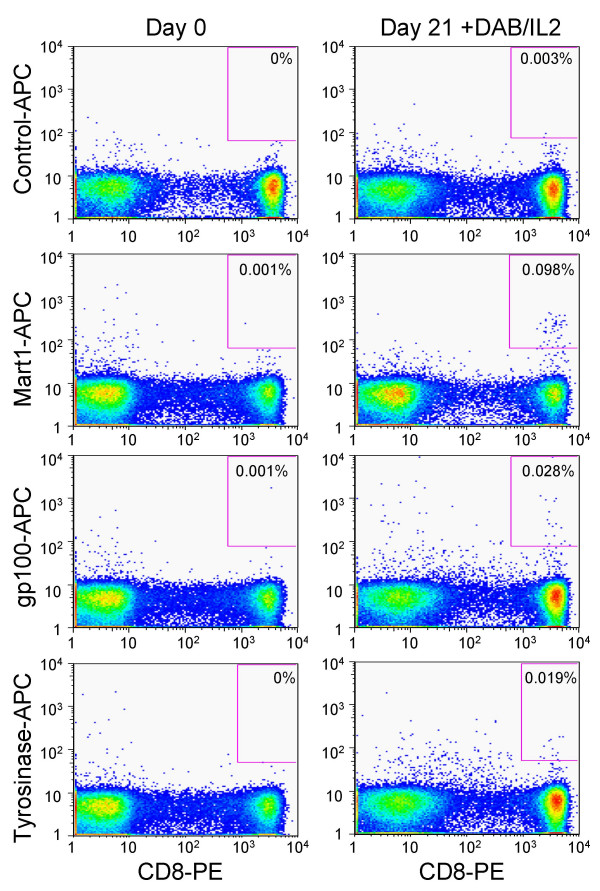
**Flow cytometric scatter plots demonstrating the *de novo *appearance of MART1-, gp100- and tyrosinase-specific CD8^+ ^T cells after one cycle of DAB/IL2**. Whole blood was collected from patient P16 during cycle one of DAB/IL2 administration just prior to (day 0) and 21 days after the first dose of DAB/IL2. The peripheral blood mononuclear cells were isolated from the whole blood by Ficoll gradient centrifugation and stained with a PE-labeled anti-CD8 monoclonal antibody and the indicated APC-labeled tetrameric HLA-A2*0201/peptide conjugates.

**Figure 6:**
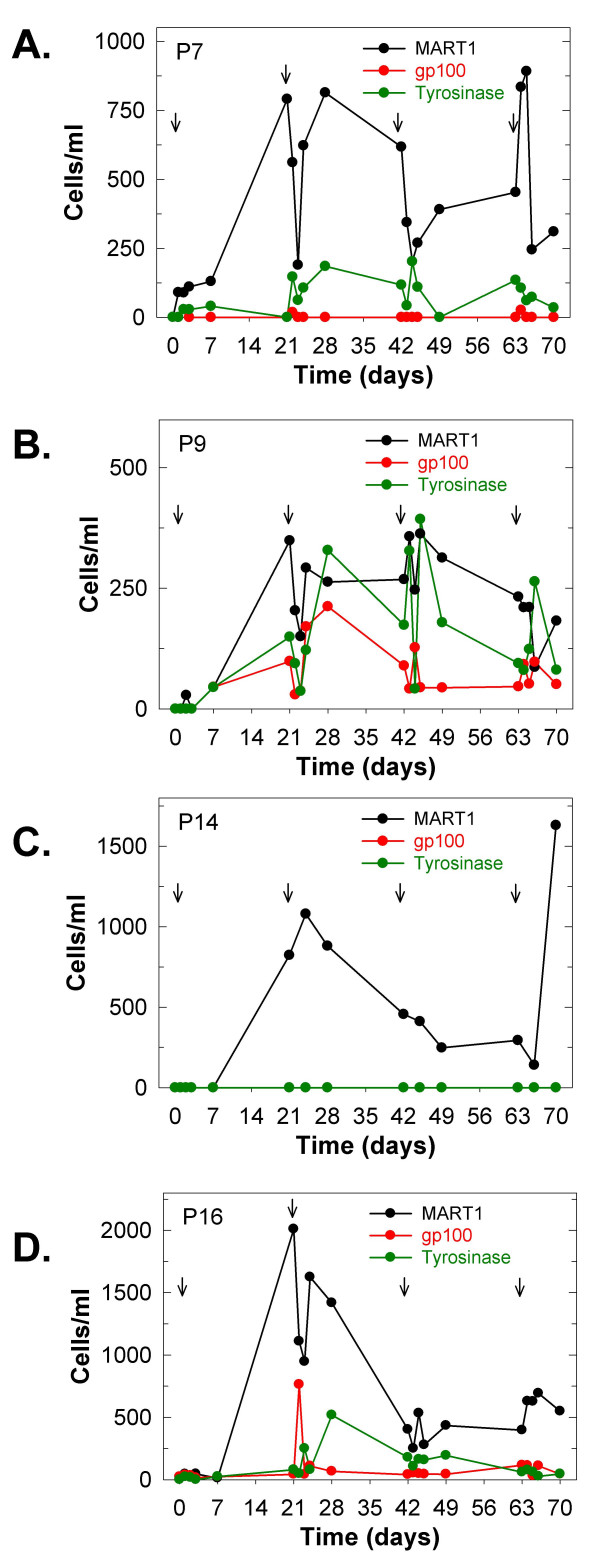
*De novo *appearance of MART1-, gp100- and/or tyrosinase-specific CD8^+ ^T cells in 4/7 HLA-A2*0201^+ ^melanoma patients after one cycle of DAB/IL2. Whole blood was collected from patients P7 (**A**), P9 (**B**), P14 (**C**) and P16 (**D**) throughout four cycles of DAB/IL2 administration (each cycle indicated by an arrow). The peripheral blood mononuclear cells were isolated from the whole blood by Ficoll gradient centrifugation and stained with a PE-labeled anti-CD8 monoclonal antibody and APC-labeled tetrameric HLA-A2*0201/MART1 (black) or gp100 (red) or tyrosinase (green) peptide conjugates. The peripheral blood concentration of the indicated melanoma antigen-specific CD8^+ ^T cells was quantified by multiplying the percentage of CD8^+^/tetramer^+ ^cells within the lymphocyte forward/side scatter gate by the absolute lymphocyte concentration determined using an automated hematology analyzer. Data are represented as averages ± standard error of the mean (n = 10 patients). Patient P14 did not develop detectable tyrosinase- or gp100-specific CD8+ T cells but the green line (tyrosinase) is concealing the red line (gp100) (C).

Seven patients expressed the HLA-A2*0201 class I MHC necessary for tetramer-based measurement of CD8^+ ^T lymphocytes specific for the melanoma antigens, MART1, tyrosinase and gp100. We did not detect CD8^+ ^T cells specific for these three melanoma peptide/MHC conjugates prior to DAB/IL2 administration in any of the examined seven patients (*data not shown*). However, we did observe the *de novo *appearance of MART1-specific CD8+ T cells in four HLA-A2*0201^+ ^patients after one cycle of DAB/IL2 as well as CD8^+ ^T cells specific for gp100 and tyrosinase in 2 and 3 patients, respectively (Figures 5 and 6). Interestingly, DAB/IL2 transiently decreased the newly detectable MART1-specific CD8^+ ^T cells in three patients at the initiation of cycles 2 and 3 (see Figures 6B–D). These data support the hypothesis that transient depletion of T cells in melanoma patients may disrupt the homeostatic control of cognate immunity and allow for the expansion of effector T cells with specificity against melanoma cells.

### DAB/IL2 decreases tumor burden in stage IV melanoma patients

**Figure 7:**
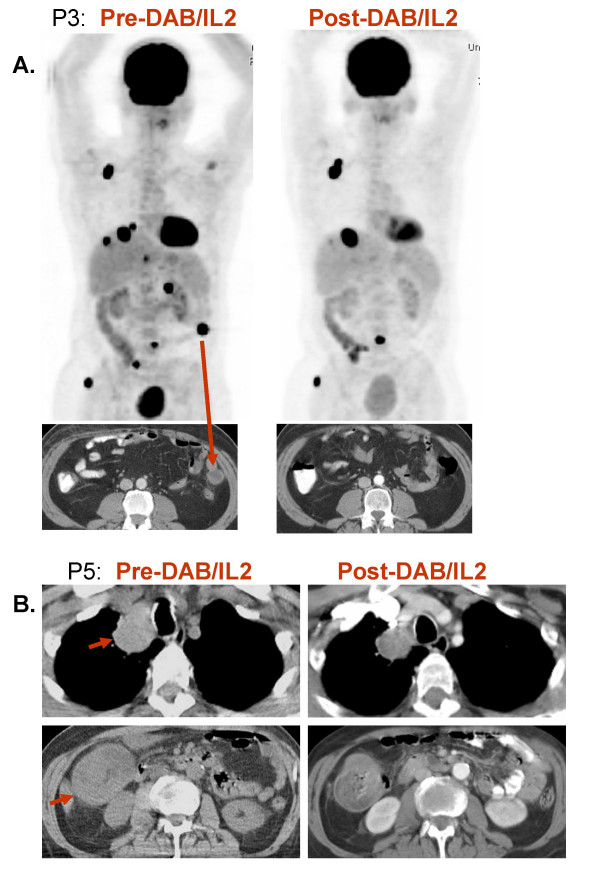
**Regression of hepatic, mesenteric and hilar melanoma metastases after DAB/IL2 administration**. **A**. Patient P3 was scanned by combination PET/CT imaging 2 weeks prior to DAB/IL2 administration (pre-DAB/IL2) and after completing four 3-week cycles of DAB/IL2 (post-DAB/IL2). The brain, heart and bladder have normal accumulations of the PET tracer ^18^F-fluorodeoxyglucose but several areas of increased metabolism consistent with melanoma metastases resolved after DAB/IL2 administration. **B**. CT imaging of patient P5 revealed a large right hilar mass and a mesenteric mass that both decreased in size after DAB/IL2 administration.

**Figure 8:**
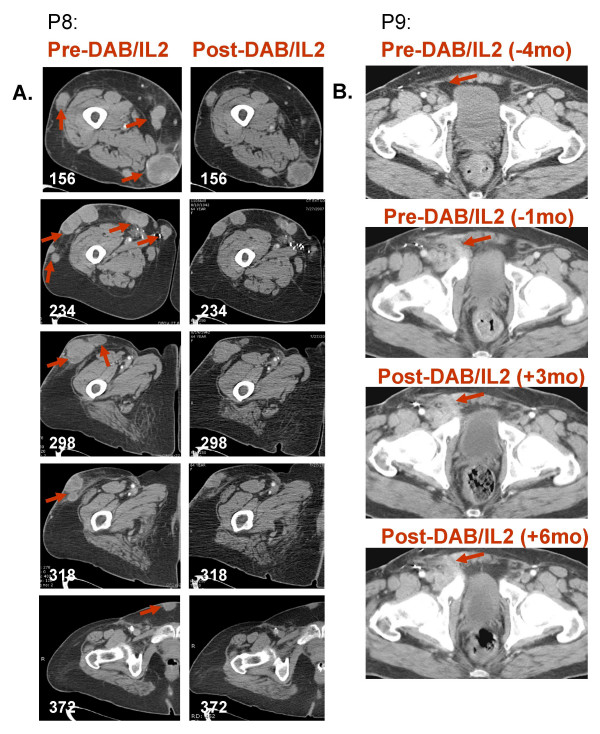
**Regression of subcutaneous, intramuscular and lymphatic metastases after DAB/IL2 administration**. **A**. The right lower extremity of patient P8 was scanned by CT imaging 3 weeks prior to DAB/IL2 administration (pre-DAB/IL2) and after completing four 3-week cycles of DAB/IL2 (post-DAB/IL2). The white numbers in the lower left corner of each image indicate the distance (mm) above the superior aspect of the patella in order to provide matched images for comparison. **B**. CT imaging of patient P9 revealed a rapidly growing right inguinal mass that decreased in size 3 months after DAB/IL2 administration. A follow-up scan, 6 months after DAB/IL2 administration, revealed no further growth.

**Figure 9:**
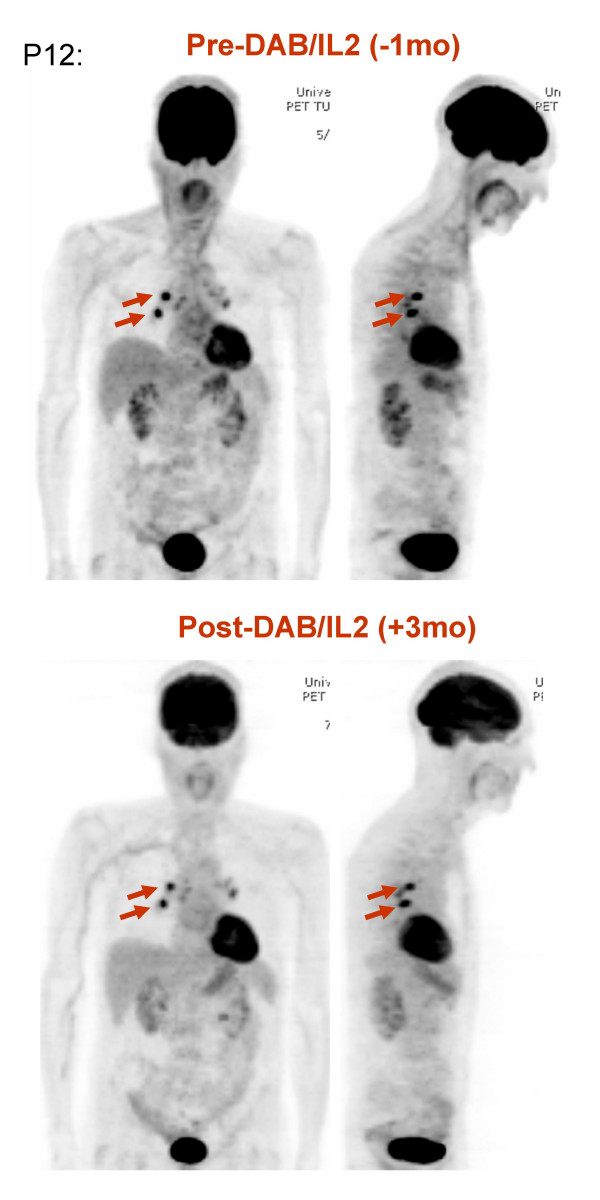
**Stabilization of two right hilar masses in a 79-year old male after DAB/IL2 administration**. PET imaging of patient P12 was conducted 1 month prior and 3 months after 2 cycles of DAB/IL2. Two discrete areas of hypermetabolism in the right hilum remained stable during this three month period.

Sixteen heavily pre-treated stage IV melanoma patients were administered 1–4 cycles of DAB/IL2 (12 μg/kg daily × four days every 3 weeks). Positron emission tomography and/or computed tomography were used to evaluate the patients' baseline tumor burden and potential responses three months from initiation of therapy. Table 1 details the characteristics of the patients and the observed responses as per RECIST criteria. We observed reductions in tumor burden in five patients and stabilization of disease in one patient. Patient P3 had developed rapidly progressing subcutaneous, hepatic and mesenteric metastases but after 4 cycles of DAB/IL2, experienced regression of 7 tumors as measured by PET/CT imaging (Figure 7A). Interestingly, the two largest metastases at baseline grew during treatment. Patient P5 experienced regression of two large melanoma metastases, a right hilar and a right colonic mass (Figure 7B). Patient P8 had several palpable subcutaneous and intramuscular metastases in her right lower extremity decrease in volume by physical exam after the first cycle of DAB/IL2. She completed four cycles of DAB/IL2, and CT imaging confirmed a decrease in the size of all metastases (Figure 8A). Patient P9 developed swelling in his right inguinal basin and CT imaging confirmed the development of a large right inguinal mass over a 3 month interval. He experienced decreased swelling after two cycles which was confirmed by CT imaging after a total of four cycles of DAB/IL2 (Figure 8B). This mass did not change in size for the next 3 months and, after its surgical resection, the patient had no further evidence of disease. The oldest patient enrolled (79 years old; patient P12), developed two right hilar metastases which became less prominent by PET imaging after two cycles of DAB/IL2 (designated Stable Disease; Figure 9).

**Table 1:** Adverse Events and Clinical Outcomes of DAB/IL2 Administration to Melanoma Patients

**ID**	**Age**	**M/F**	**Stage**	**Cycles**	**Adverse Events (grade)**	**Outcome(s)**	HLA-A2* 0201	**CD8+ T Cells**
P1	50	F	IV	2	Erythema (1)	Progressive disease	-	Not applicable
P2	78	M	IV	3	Weakness (1)	Progressive Disease	-	Not applicable
P3	58	M	IV	4	None	Mesenteric & Hepatic Regressions (PR)	-	Not applicable
P4	66	F	IV	1	None	Progressive Disease	-	Not applicable
P5	72	M	IV	2	Pain at tumor site (1)	R. Hilar and Colon Regressions (PR)	-	Not applicable
P6	63	M	IV	2	None	Progressive Disease	-	Not applicable
P7	67	M	IV	4	None	Progressive Disease	+	MART1^+^Tyr^+^
P8	58	F	IV	4	None	IM & SC Regressions (PR)	-	Not applicable
P9	46	M	IV	4	None	Inguinal Regression	+	MART1^+^g100^+ ^Tyr^+^
P10	35	F	IV	3	None	Progressive Disease	+	Negative
P11	54	M	IV	2	None	Progressive Disease	+	Negative
P12	79	M	IV	2	Dermatitis (2)	Stable Disease	-	Not applicable
P13	72	M	IV	1	Dehydration (2)	Progressive Disease	-	Not applicable
P14	61	M	IV	4	Vitiligo (1)	Pulmonary, Hepatic, LN & SC Regressions (PR)	+	MART1^+^
P15	46	F	IV	4	None	Progressive Disease	+	Negative
P16	68	F	IV	4	Arthritis (2) Dermatitis (1)	Progressive Disease	+	MART1^+^g100^+ ^Tyr^+^

**Figure 10:**
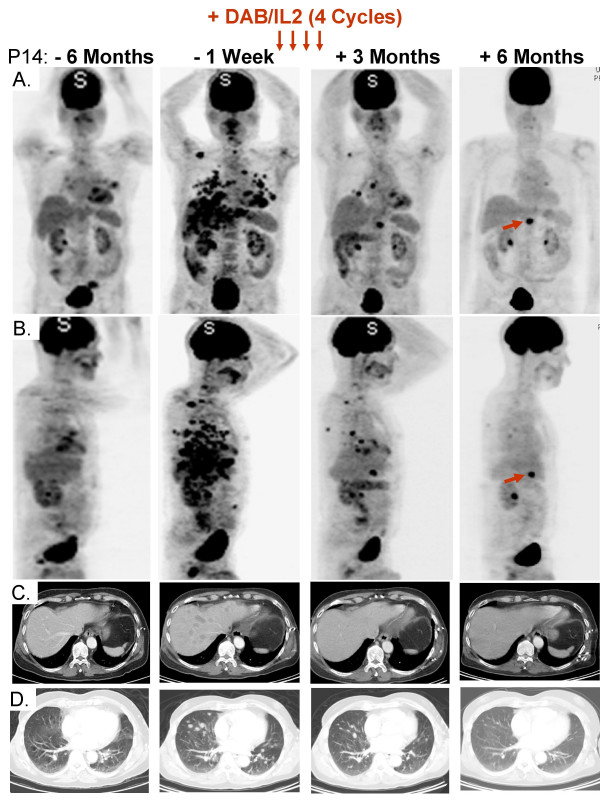
**Near complete response of widespread visceralmelanoma metastases after 4 cycles of DAB/IL2**. **A**. Anterior/posterior views, PET. **B**. Lateral views, PET. **C**. CT imaging, liver. **D**. CT imaging, lungs. Combined PET/CT imaging of patient P14 revealed rapid progression of multiple melanoma metastases in the liver, both lungs, lymph nodes and the subcutaneous compartment (compare -6 months to -1 week). After 4 cycles of DAB/IL2, the liver metastases completely resolved and the lung metastases markedly regressed (compare -1 week to +3 months). Three months after completion of DAB/IL2, the residual lung metastases had completely resolved but a single enlarged peri-aortic lymph node persisted (red arrow). The increased ^18^F-fluorodeoxyglucose uptake in the brain, bladder and both kidneys are due to normal metabolism and are not reflective of metastases.

**Figure 11:**
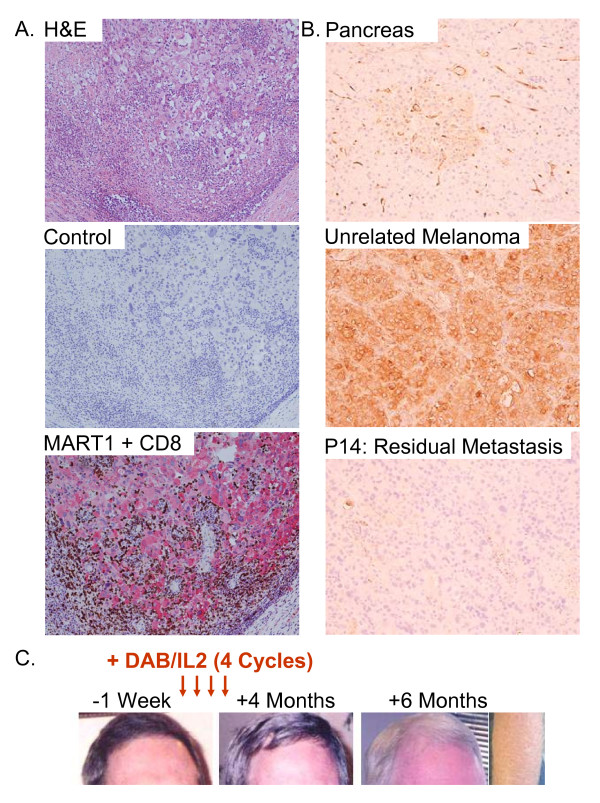
**CD8^+ ^T cell infiltration of residual HLA-A, B and C negative melanoma and evidence for vitiligo after DAB/IL2 administration**. The residual peri-aortic mass in patient P14 was resected, formalin fixed and embedded in paraffin. **A**. Hematoxylin/eosin (H&E) staining of the mass revealed a mononuclear infiltrate that was confirmed to include CD8^+ ^T cells by double immunohistochemistry using an anti-CD8 antibody (brown) and an anti-MART1 antibody (red). The counter stain used in the immunohistochemistry was hematoxylin and the control consisted of no primary antibody. **B**. HLA-A, B or C expression by cells in the pancreas (top; positive control), an unrelated melanoma metastasis (middle; positive control) and the residual peri-aortic melanoma metastasis resected from patient P14 as determined using a monoclonal antibody specific for a non-polymorphic portion of these HLA molecules. **C**. Photographs of patient P14's hair before and after DAB/IL2 administration revealed the complete loss of pigmentation.

Patient P14 developed widespread melanoma involving the lungs, liver, subcutaneous compartment and adrenal glands over a six month period (Figure 10). Clinically, he was suffering from appetite and weight loss, fatigue, nausea and shortness of breath. After four cycles of DAB/IL2, PET/CT imaging revealed the complete regression of all hepatic metastases and the majority of pulmonary metastases (Figure 10). Follow-up PET/CT imaging three months after completion of the fourth cycle of DAB/IL2 demonstrated resolution of the residual pulmonary metastases but a persistently enlarged peri-aortic lymph node. Surgical resection of this residual metastasis was conducted and H&E staining revealed a mononuclear infiltrate within a metastatic melanoma. Double immunohistochemistry for the melanoma protein MART1 and CD8 demonstrated that the melanoma cells (red staining) were surrounded by infiltrating CD8^+ ^T cells (brown staining; Figure 11A) but not CD4^+ ^T cells (*data not shown*). These remaining melanoma cells were completely devoid of HLA-A, B or C expression as determined using a monoclonal antibody specific for a non-polymorphic portion of these HLA molecules (Figure 11B; compare positive controls, pancreas and unrelated melanoma, to P14 residual metastasis). Coincident with this objective response, the patient reported decreased pigmentation in his hair and skin consistent with the development of vitiligo, an autoimmune disease against melanocytes (Figure 11C).

We were surprised by the high partial response rate in these 16 patients and postulated that DAB/IL2 may exhibit direct cytotoxic effects against human melanoma cells. However, exposure of DAB/IL2 to proliferating human melanoma cells *in vitro *at a concentration 15-fold higher than the obtainable peak plasma concentration of DAB/IL2 in humans (0.3 μg/ml) had no effect on cell viability or proliferation (0.05–5 μg/ml × 48 hours; vehicle control, 7.12 ± 0.13 × 10^5 ^cells; + 5 μg/ml DAB/IL2, 7.35 ± 0.37 × 10^5 ^cells; *p *value = 0.444).

## Discussion

Melanoma incidence has risen by 25–31% over the last decade and is now the 5^th ^most common cancer in men and the 6^th ^most common cancer in women [1,17]. Melanoma causes a disproportionate mortality in young and middle-aged individuals and, as such, displays one of the highest "loss of potential life" rates among the adult-onset cancers (18.6 years per melanoma-related death) [1]. In the United States, over 8000 adults die of melanoma annually, and 84% of melanoma patients with distant metastases succumb to their disease within 5 years of diagnosis [1].

The treatment options for patients with metastatic melanoma are limited to palliation or to aggressive therapy with high dose IL-2 or biochemotherapy using cisplatin, vinblastine, dacarbazine, IL-2 and interferon α-2b. The response rate to high dose IL-2 is low (16%) but durable cures have been observed in approximately 6–10% of the patients that can tolerate the systemic toxicity (*i.e*. hypotension, capillary leak syndrome, sepsis and renal failure) [3,4]. Although biochemotherapy has been reported to yield a 35–50% partial response rate and up to a 20% complete response rate, median survival duration is only 12.2 months [17,18]. Early published reports of clinical trials of humanized anti-CTLA4 monoclonal antibodies have indicated a 10–20% partial response rate in melanoma patients [19]. In the current study, we observed a 31% partial response rate after treatment with DAB/IL2 (5/16 patients) which is clinically significant given the low toxicity of this agent. Importantly, the majority of patients who are treated with high dose IL-2, biochemotherapy and/or anti-CTLA4 ultimately experience progression and few efficacious alternative treatments are currently available.

We found that transient depletion of CD4^+ ^and CD8^+ ^T cells in melanoma patients via targeting of IL-2 receptor-expressing cells resulted in T cell repopulation in the peripheral blood and the *de novo *appearance of CD8^+ ^T cells with specificity for melanoma antigens (in 4/7 HLA-A2*0201 patients). We had anticipated that the detection of peripheral blood MART1-, gp100- and tyrosinase-specific CD8^+ ^T cells in these HLA-A2*0201^+ ^patients might correlate with tumor regressions. The three HLA-A2*0201^+ ^patients who did not develop any detectable MART1-, gp100- and tyrosinase-specific CD8^+ ^T cells also did not experience regression of their melanoma metastases (Table 1). However, we observed the regression of melanoma metastases in only 2/4 HLA-A2*0201^+ ^patients who developed melanoma antigen-specific CD8^+ ^T cells (Table 1). We can only speculate that the two patients who developed melanoma antigen-specific CD8^+ ^T cells but did not experience tumor regressions may have melanomas that express low class I MHC or effector CD8^+ ^T cells that are compromised by low affinity T cell receptors and/or the tumor microenvironment. Importantly, the peptide/MHC tetramers used in this study can only detect a miniscule fraction of the possible CD8^+ ^T cells that have specificity for MART1, gp100, tyrosinase or other melanoma antigens.

Intriguingly, patients P3 and P14 experienced the regression of multiple metastastic melanomas simultaneously with the persistence and even growth of other metastatic melanomas (*i.e*. a mixed response). The residual peri-aortic mass in patient P14 was confirmed to express the melanoma antigen, MART1, and this patient developed peripheral blood MART1-specific CD8^+ ^T cells within 21 days of transient T cell depletion. Despite immunohistochemical evidence that CD8^+ ^T cells appeared to surround the MART1^+ ^melanoma cells, this residual metastatic melanoma was not cleared. Interestingly, the melanoma cells did not express the class I MHC proteins HLA-A, B or C which may partly explain the lack of regression of this particular metastasis. We suspect that differences in melanoma antigen expression and/or additional tumor immunoevasion tactics also may explain such differential anti-tumor effects within a single host and future studies will be directed at further examination of the phenotypes of melanoma cells and infiltrating immune cells in growing and regressing melanomas within a single host.

DAB/IL2 administration transiently decreased CD4^+^CD25^-^, CD4^+^CD25^+^, CD4^+^CD25^HI^Foxp3^-^, CD4^+^CD25^HI^Foxp3^+^, CD8^+ ^T cells and, in certain patients, melanoma antigen-specific CD8^+ ^T cells. These data suggest that DAB/IL2 is not selectively cytotoxic to T regulatory cells which may be due, in part, to the high IL-2 receptor expression of activated effector T cells. We found that all examined T cell subsets repopulated the peripheral blood and presume that this repopulation is due either to a proliferative expansion or re-trafficking of T cells from lymph nodes. Interestingly, CD4^+ ^or CD8^+ ^T cell depletion in mice has been found to cause a proliferative expansion of the residual T cells that restores the original T cell pool size [20]. This peripheral expansion has been termed homeostatic proliferation and can prevent the induction of tolerance to transplanted organs and cause anti-tumor responses against melanomas and colon cancer in mice [20-22]. Although the mechanisms for these effects are not well established, homeostatic proliferation of CD4^+ ^and CD8^+ ^T cells is, in part, driven by MHC/peptide recognition. We postulate that transient T cell depletion in cancer patients may cause a rebound expansion of T cells with a shifted TCR repertoire that includes increased melanoma antigen-specific CD8^+ ^T cells. On-going studies that examine the relative effects of Treg depletion and transient total T cell depletion on melanomas in mice and the consequences of adding back Treg cells or total lymphocytes on these effects should improve our understanding of the precise mechanisms for the observed tumor regressions in melanoma patients.

The effect of DAB/IL2 on both peripheral blood Treg cell concentration and tumor burden has been previously examined in 4 patients with metastatic breast, lung or ovarian cancer (single infusion; 9 μg/kg or 12 μg/kg) [23]. Prior to infusion, the mean Treg cells/mm^3 ^was found to be 126 (26.8% of total CD4+ Tcells) and, after DAB/IL2 infusion, the mean Treg cells/mm^3 ^was reduced to 78 (19.0% of total CD4+ Tcells) [23]. A patient with Stage IIIC, relapsed ovarian cancer received the highest dose of DAB/IL2 (12 μg/kg) and experienced a marked reduction in the ovarian cancer marker, CA-125, four weeks after a single infusion (from 121 U/ml to 38 U/ml) [23]. This patient was then administered 6 additional doses of DAB/IL2 weekly (12 μg/kg) and, two months after the final dose, a PET/CT fusion scan revealed a dramatic reduction in metastatic burden. In a second study, twelve stage IV melanoma patients were administered DAB/IL2 at a lower dose (9 μg/kg) and a higher dose (18 μg/kg) daily × 5 days every three weeks [24]. The investigators reported that none of the patients experienced an objective clinical response and that the expression of the Treg-specific transcription factor Foxp3 and the suppressive ability of CD4^+^CD25^HI ^cells did not decrease significantly. The disparity in clinical results between this study and the current study of melanoma patients may be due to differences in: (i) the dosing (9 or 18 versus 12 μg/kg); (ii) the duration of treatment in each cycle (5 daily versus 4 daily doses per cycle); (iii) the low sample size in both studies; (iv) the extent of disease in the examined human subjects; (v) the methods used to measure T regulatory cells (real-time PCR for Foxp3 versus three color flow cytometry for CD4^+^CD25^HI^Foxp3^+ ^cells); and (vi) the extent of prior immunotherapeutic treatments that had been administered to the patients in each study (*e.g*. IL-2). Importantly, in a recent study, DAB/IL2 was found to significantly reduce the peripheral blood Treg cells in metastatic renal cell carcinoma patients and to abrogate Treg-mediated immunosuppressive activity *in vivo *[25]. This study also demonstrated that DAB/IL2-mediated depletion of Tregs followed by vaccination with RNA-transfected DCs increased the activation of tumor-specific T cell responses compared to vaccination alone [25].

## Conclusion

In conclusion, we have demonstrated that T cell depletion with DAB/IL2 in melanoma patients is followed by a T cell repopulation of the peripheral blood, the *de novo *appearance of CD8^+ ^T cells specific for melanocyte differentiation antigens and regression of melanoma metastases. This single site, investigator-initiated phase II clinical trial has been expanded to provide further evidence for the efficacy of DAB/IL2 against melanoma. Future clinical studies that are conceptually appealing include an examination of the efficacy of DAB/IL2 in several cancer types and the potential utility of combination therapy with vaccines or other immune enhancing agents such as interferon-α. Last, we anticipate that the limited or pulsed administration of alternative T cell depleting agents may prove useful for the activation of cognate immunity against neoplastic cells in cancer patients.

## Abbreviations

The abbreviations used in this manuscript incuded: Treg, T regulatory cells; MHC, Major histocompatibility complex; DAB/IL2, Denileukin diftitox or Ontak; CTLA-4, Cytotoxic T lymphocyte antigen-4; CTCL, Cutaneous T cell lymphoma; IL-2, Interleukin 2; MART-1; Melanoma-associated antigen recognized by T cells; PET/CT, Positron emission tomography and computed tomography; CR, Complete response; PR, Partial response; PD, Progressive disease.

## Competing interests

The author(s) declare that they have no competing interests.

## Authors' contributions

All authors have read and approved the final manuscript. The specific contributions of each author are: MAR conducted flow cytometric experiments coordinated entire study and prepared all figures; ALC assisted with the flow cytometric experiments; ST collected human tissues and coordinated immunohistochemical analysis; BT provided research nursing support related to the conduct of the clinical trial; KG provided clinical nursing support related to the conduct of the clinical trial; HG provided regulatory support related to the conduct of the clinical trial; DC conducted flow cytometry; SCL conducted immunohistochemistry; KMM resected surgical specimens for immunohistochemistry; DMM co-conducted the clinical trial and assisted with imaging analyses; JC conceived, designed and directed the entire study, interpreted all data and wrote the manuscript.
